# Low‐dose benzodiazepine receptor agonists may be completely replaced by lemborexant at about the same dose

**DOI:** 10.1002/pcn5.32

**Published:** 2022-08-09

**Authors:** Kensuke Usui, Yu Fujii, Kouji Okada, Eiji Suzuki

**Affiliations:** ^1^ Division of Clinical Pharmaceutics and Pharmacy Practice Tohoku Medical and Pharmaceutical University Sendai Japan; ^2^ Department of Pharmacy Tohoku Medical and Pharmaceutical University Hospital Sendai Japan; ^3^ Division of Psychiatry Tohoku Medical and Pharmaceutical University Sendai Japan

## Abstract

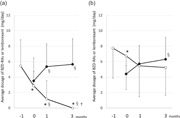

## AIM

Because of problems such as tolerance, addiction, abuse, and falls, many attempts have been made to reduce the prescription dose of benzodiazepine receptor agonists (BZD‐RAs).^1^ However, BZD‐RAs remain the first‐line hypnotic drug in Japanese clinical practice.^2^ The US Food and Drug Administration issued a warning about the risks of BZD‐RAs in 2020.^3^

A phase III clinical study on lemborexant, a recently developed orexin receptor blocker, stated that lemborexant therapy showed better efficacy than BZD‐RAs for objectively measured sleep onset and maintenance.^4^ Moreover, another report stated that lemborexant significantly improved subjective sleep latency in the first week of administration compared to suvorexant, an existing orexin receptor blocker.^5^ Therefore, lemborexant was regarded as a candidate drug for switchover from BZD‐RAs. Recently, it was reported that the dose of BZD‐RAs used could be reduced using lemborexant in clinical practice.^6^, ^7^ Hence, this study aimed to characterize patients in whom BZD‐RAs could be completely or not completely replaced by lemborexant at our facility.

## METHODS

Among patients who were already taking BZD‐RA hypnotics at our facility, those who were prescribed lemborexant from July 2020 to March 2021 were investigated, and the changes in lemborexant and BZD‐RAs were retrospectively evaluated. Patients were taking various BZD‐RAs, thus individual BZD‐RAs doses were expressed as equivalent doses of diazepam.^8^ The group of patients who completely stopped BZD‐RAs 3 months after starting lemborexant was defined as the discontinuing group (DG), whereas the group of patients who continued taking BZD‐RAs was defined as the continuing group (CG). Both groups were compared using a *t*‐test. The Mann–Whitney test was used to compare the prescribed dosages of independent groups. A one‐sample Wilcoxon signed‐rank test with Bonferroni correction was also performed, and the data are expressed as mean ± standard deviation.

## RESULTS

Our sample included 69 individuals (52.1 ± 16.9 years old), of whom 44 were women, 21 were hospitalized, and 40 experienced concurrent psychiatric disorders (16 had depression, 14 had bipolar disorder, 5 had schizophrenia, and 5 had other diseases). After 3 months, 63 of the 69 (91.3%) patients continued taking lemborexant. Two and four patients discontinued lemborexant because of nightmare side effects and insufficient efficacy, respectively.

**Figure 1:**
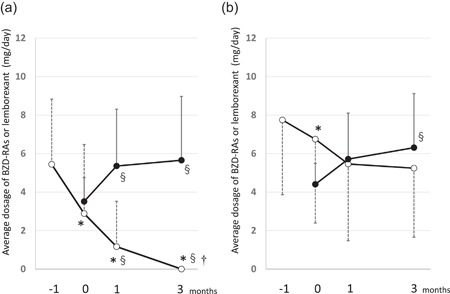
Changes in the prescribed dosage of BZD‐RAs and lemborexant. The change in prescription dosage during the switch from BZD‐RA to lemborexant. Data of 63 patients who had been using BZD‐RAs but had lemborexant prescribed and whose course could be confirmed up to 3 months later. (a) The group in which BZD‐RAs could be completely discontinued (discontinuing group, *n* = 42). (b) The group in which BZD‐RAs were continued (continuing group, *n* = 21). Open circles indicate the average dosage of BZD‐RAs. The amount of BZD‐RA is shown as the diazepam equivalent dose. Closed circles indicate the average dosage of lemborexant. The numbers −1, 0, 1, and 3 shown on the horizontal axis indicate 1 month before switching to lemborexant, at the time of switching, and 1 and 3 months after switching, respectively. *p < 0.05 versus −1 month (Wilcoxon signed rank test with Bonferroni correction), ^§^ *p* < 0.05, versus 0 months (Wilcoxon signed rank test with Bonferroni correction), ^†^ *p* < 0.05 versus 1 month (Wilcoxon signed rank test with Bonferroni correction).

Figure 1 shows the time‐course of diazepam‐equivalent doses of BZD‐RAs and lemborexant in the DG (a) and CG (b). The numbers of patients in the DG and CG were 42 (66.7%) and 21 (33.3%), respectively. Furthermore, the BZD‐Ras dose before the initiation of emborexant in the DG was significantly lower than that in the CG (5.4 ± 3.4 vs. 7.8 ± 3.9 mg). The dose of BZD‐RAs was reduced during the 3 months in the CG but was significantly higher than that in the DG (5.4 ± 3.4 vs. 2.5 ± 4.0 mg).

In the DG, 5.4 ± 3.4 mg of BZD‐RAs was switched to 5.7 ± 3.3 mg of lemborexant, and therefore, the diazepam equivalent dose of lemborexant was calculated to be 0.96 mg. No statistical differences were observed between the two groups in gender, presence or absence of psychosis, age, and hospitalization (inpatient or outpatient). In th eDG, the dose of BZD‐RAs significantly decreased from initiation to 1 month post‐intervention (*p* < 0.0001) and from 1 to 3 months post‐intervention (*p* < 0.0001); in contrast, in the CG, the dose of BZD‐RAs decreased in the former period (*p* = 0.044) but did not decrease (*p* > 0.9) in the later period.

## DISCUSSION

Our results suggest that a relatively high proportion (66.7%) of patients can completely switch from BZD‐RAs to lemborexant, consistent with a previous study^6^ that reported that 58.4% of patients could completely switch. Our study suggests that the predictors of the complete replacement of BZD‐RAs with lemborexant include the low dose of BZD‐RAs administered and the reduced dose of BZD‐RAs from 1 to 3 months post‐intervention after lemborexant initiation. In a previous report, the average dosage of BZD‐RAs administered before switching was 4.6 ± 2.8 mg and that of lemborexant after switching was 6.2 ± 2.2 mg.^6^ In contrast, in this study BZD‐RAs were switched to lemborexant at about the same dose in the DG. Although it is unclear why the dose of lemborexant for BZD‐RAs was higher in the previous study than in this study, one possibility is that the previous study included patients who could not completely switch from BZD‐RAs to lemborexant.

Although this study has a large methodological limitation because it is a retrospective survey of the actual clinical prescriptions of one institution, we think it is valuable because it shows the predictors of a successful switch from BZD‐RAs to lemborexant.

## AUTHOR CONTRIBUTIONS

Kensuke Usui designed the study, performed the statistical analyses, analyzed and interpreted the data, and drafted the manuscript. Yu Fujii collected the data and were involved in the manuscript drafting and editing. Kouji Okada and Eiji Suzuki performed the drafting and editing and contributed to supervision. All authors read and approved the final manuscript.

## CONFLICT OF INTEREST

Eiji Suzuki received research funding from Eisai Co., Ltd. and Shionogi & Co., Ltd.

## ETHICS APPROVAL STATEMENT

This study was implemented after obtaining the approval of the Tohoku Medical and Pharmaceutical University Hospital's Ethics Committee (Approval No.: 2021‐2‐001).

## PATIENT CONSENT STATEMENT

Informed consent was not directly sought from the study subjects as this was a retrospective, observational study. However, we published information on the study on the hospital website and, in the place of informed consent, we guaranteed “opt‐out” option for the patients to overrule the use of their data in the research.
